# The Spatial and Temporal Deployment of Voluntary Attention across the Visual Field

**DOI:** 10.1371/journal.pone.0006716

**Published:** 2009-08-21

**Authors:** Guilhem Ibos, Jean-René Duhamel, Suliann Ben Hamed

**Affiliations:** Centre de neuroscience cognitive, CNRS - Université Claude Bernard Lyon 1, Bron, France; Istituto di Neurofisiologia, Italy

## Abstract

Several studies have addressed the question of the time it takes for attention to shift from one position in space to another. Here we present a behavioural paradigm which offers a direct access to an estimate of voluntary shift time by comparing, in the same task, a situation in which subjects are required to re-engage their attention at the same spatial location with a situation in which they need to shift their attention to another location, all other sensory, cognitive and motor parameters being equal. We show that spatial attention takes on average 55 ms to voluntarily shift from one hemifield to the other and 38 ms to shift within the same hemifield. In addition, we show that across and within hemifields attentional processes are different. In particular, attentional spotlight division appears to be more difficult to operate within than across hemifields.

## Introduction

Attention is a psychological construct representing the mechanisms by which the selection and processing of visual information is facilitated [1]. A major question in the study of selective visual attention is understanding how voluntary endogenous attention moves from one location to another [2]–[4]. In the present study, we focused specifically on the temporal dynamics of voluntary attention control both within and across visual hemifields. Several studies have tried to estimate the time it takes for endogenous attention to shift from one spatial location to another. The contribution of Sperling and his collaborators in the 1980's was very important in this respect [5], [6]. Indeed, to address this question, they developed a dual-stream rapid serial visual presentation (RSVP) paradigm in which subjects were required to maintain central fixation on a stream of numeral stimuli while at the same time monitoring a peripheral stream of letter stimuli in order to detect an embedded target letter. On detection of the target letter, they were asked to shift their attention to the numeral stream and report the four first numerals they perceived as concomitant or directly following the detected letter. The earliest number detected was thus the temporal marker of the voluntary shift of attention from the letter stream to the numeral stream, which was estimated in the range of 300 to 400 ms. More direct measures of the dynamics of attentional deployment estimate that voluntary shifts take place 150 to 200 ms from the instruction [5], [6]. This result has been reproduced by other studies [7], [8]. However, as mentioned by Kinchla “… this “attention reaction time” ostensibly includes the time to recognize the target letter, as well as the time to switch attention and the two are hard to separate” [9]. Other possible cognitive operations can take place during this time interval, including shifting between the analysis of numerals and the analysis of letters, and interfere with target detection such as memorization of digits [10], [11]. Indirect estimates of attention shift times can be derived from the visual search literature [12]–[15]. In particular, Wolfe et al showed that 50 ms per item is the minimal possible dwell time of attention for a subject to perform a visual search task [12], [13]. Dwell time in this context of serial search can be considered as the sum of stimulus perceptual analysis plus the time needed by attention to move from one stimulus to the next, thus providing an upper bound of the minimal time needed by the attentionnal spotlight to shift.

In order to obtain a more direct evaluation of the time needed by voluntary spatial attention to shift independently of any additional perceptual, cognitive or motor parameter, we have used a modified version of the dual stream RSVP paradigm of Yantis et al. [16]. Subjects were required to maintain central fixation while monitoring one of two peripheral streams in search of a target image which they had to detected as fast as possible by a key press. Two types of instruction cues embedded in the initially monitored stream cued the subject as to whether the target would appear within this stream (*stay* instruction) or within the other stream (*shift* instruction). We hypothesized that a comparison of target detection reaction times on the shift and on the stay instruction should give us a direct measure of voluntary attention shift times. We also studied whether the time course of attentional allocation varied differently on attentional engagement at a new location (on *shift* instruction) as opposed to attentional re-engagement at the same location (on *stay* instruction). Finally, we positioned the streams both in the same and in different hemifields in order to investigate whether the spatial and temporal dynamics of attention was dependent on the position of the shift vector in the visual field.

## Methods

### Subjects

All experimental procedures were approved by the Ethics Committee of the Université Claude Bernard Lyon 1, and subjects gave their written informed consent. 11 subjects participated in the first experiment and 10 in the second experiment (22 to 28 years old). All subjects had normal or corrected to normal vision. All subjects were included in the study except one whose performance in the two-hemifields configuration experiment was not significantly different from chance.

### Task

In order to study the spatial and temporal dynamics of shifting and re-engaging visual covert selective attention, we designed a cued version of a dual peripheral stream Rapid Serial Visual Presentation (RSVP) task. In this task, subjects must detect the appearance of a target image in one of two streams and report it as quickly as possible by a key press. The subjects' attention is initially oriented to one of the streams, in which an embedded cue image predicts with a given probability whether the target will subsequently appear in the currently attended stream or alternatively in the other stimulus stream.

#### General task configuration

**Figure 1:**
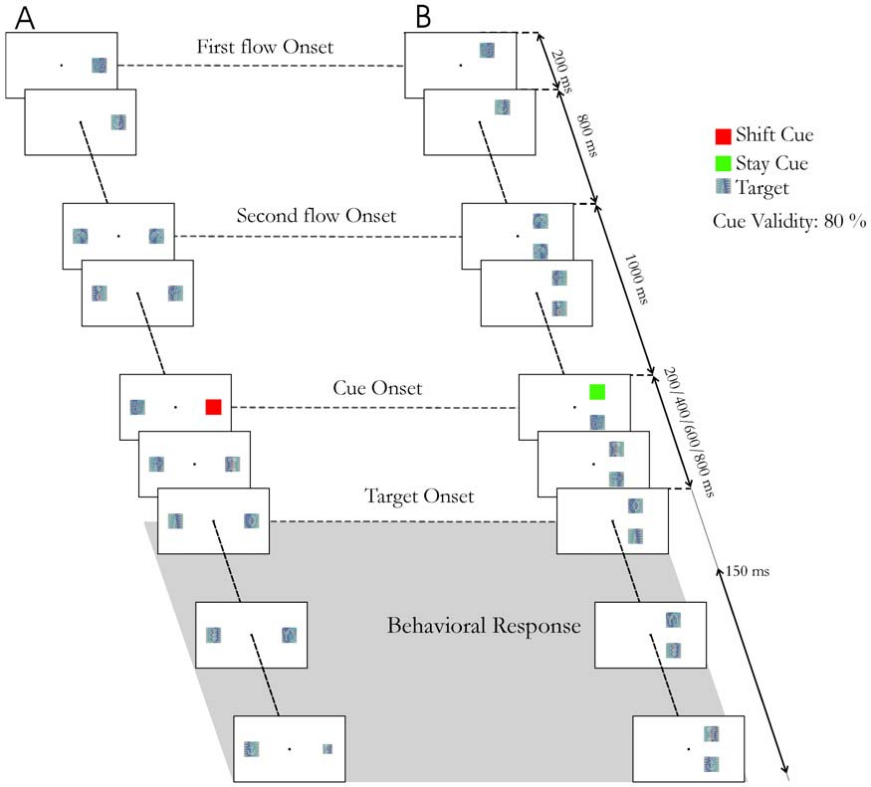
Task description. RSVP sequence for A) the two-hemifields configuration (case of a trial in which subjects are cued to shift their attention) and B) the one-hemifield configuration (case of a trial in which subjects are cued to maintain their attention on the same stream).

**Figure 2:**
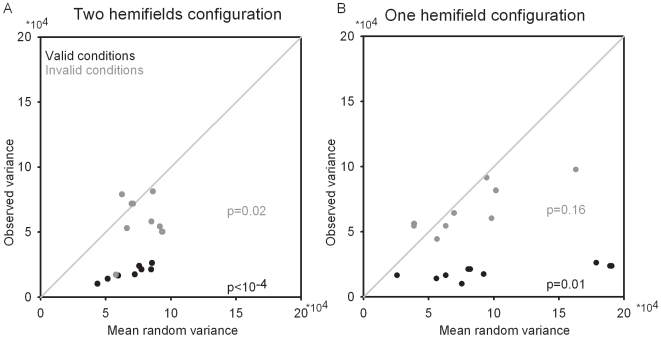
Observed reaction times distribution versus simulated reaction times distribution drawn from a uniform distribution in (A) the two-hemifields configuration and in (B) the one-hemifield configuration. Valid trials (black dots) and invalid trials (gray dots) are considered separately. P-values are indicated in corresponding colors. STD stands for standard deviation. See text for methodological details.

Subjects are required to hold their gaze on a central fixation point throughout the trial (see eye position control below). 500 ms after fixation onset, a rapid succession of 200 ms visual items (distractors), with no intervening blanks, begins at one of two possible locations on the screen (figure 1A). This stream of stimuli will be called the *first stream* and subjects are instructed to maintain their attention on this stream. 1000 ms (i.e. 5 stimuli) later, a *second stream* appears at the second location. From this point on, stimuli on both streams are presented synchronously. 600 to 800 ms (3–4 stimuli) after the onset of the second stream a cue image appears in the first stream. The cue indicates where the target will appear with a probability of 80%. We refer to the cue indicating that the target will appear in the second stream as the *Shift cue*, and the cue indicating that the target will appear in the first stream as the *Stay cue*. If the location of the target in the streams matches (resp. doesn't match) the cued instruction, then the target is called a *valid target* (resp. *invalid target*). Invalid trials are trials on which the target appears at an unexpected location, i.e. in the second stream following a *Stay cue* or in the first stream following a *Shift cue*. The target can appear at different time intervals from the cue (cue to target onset asynchronies, CTOAs: 200 ms, 400 ms, 600 ms and 800 ms). A target is thus present in all trials. The task of the subjects is to detect the embedded target and respond to it by a key press as fast as possible. Trials on which reaction times are shorter than 200 ms (anticipations) are considered as error trials. No subject was found to respond systematically, independently from target presentation (see figure 2 for a confirmation).

First stream position, cue type, target validity and CTOA were pseudorandomly distributed throughout the experimental session. For each subject, 40 trials (32 valid, 8 invalid) were recorded for a given cue type, a given cue position and a given CTOA. Two different spatial configurations were tested:

#### A two-hemifields configuration

In this configuration, the first stream of stimuli appeared on the horizontal meridian, 10° to the left or to the right of the fixation point. One second later, the second stream appeared opposite to the first stream with respect to the fixation point. Right or left location of the first stream was randomized across the trial sequence. This configuration was tested on 11 subjects (aged between 22 and 28 years old). *A one-hemifield configuration*. In this configuration, the first stream of stimuli appeared at an eccentricity of 10° in the upper right or lower right part of the visual display (at 7°×7° or 7°×−7° from the central fixation point). One second later, the second stream appeared opposite to the first stream with respect to the horizontal meridian. Upper or lower location of the first stream was randomized across the trial sequence. This configuration was tested on 10 subjects (aged between 22 and 28 years old).

### Stimuli

All stimuli were bitmaps. The central fixation point was a white square of 0.1° of visual angle. The Shift cue was a red square, the Stay cue was a green square. The target and the distractors were clipart images from Microsoft Word XP™ (Figure 1B). The size of all stimuli (cues, distractors and target) was then adjusted to match 1° of visual angle when presented at 10° of eccentricity from the fixation point. This configuration resulted in an average target detection performance equal to 66%. Mean distractors' luminance was of 20.9 cd/m^2^. Target luminance was equal to 21.1 cd/m^2^ and not distinguishable from that of the distractors. The shift cue had a luminance of 16.7 cd/m^2^ while the stay cue had a luminance of 9.3 cd/m^2^. Both luminance [17] and chromatic contrast [18] have been shown to affect reaction times. These studies predict in particular reaction times trends between *Stay* and *Shift* trials opposite to those described here, suggesting that the observations provided therein do not apply to our experimental context. In particular, while their measures were carried out for foveal stimuli, our cues are presented at 10° of eccentricity.

### Eye position

Eye position was controlled with an ISCAN© video-eye tracker (ISCAN, Inc, Burlington, MA, USA). The camera was placed in front of the subject below the screen so as to track the director eye. Eye position was calibrated for every subject at the beginning of each session, using the ISCAN calibration utility. Subjects were required to maintain fixation throughout the trial within a window of 1.5°. Breaking fixation resulted in the interruption of the ongoing trial. Interrupted trials were presented anew to the subject during the experimental session.

### Experimental procedure

This experiment was built and controlled using Cogent 2000 developed by the Cogent 2000 team at the FIL and the ICN and Cogent Graphics developed by John Romaya at the LON at the Wellcome Department of Imaging Neuroscience. Stimuli were displayed on a 17” CRT monitor with a 1024×768 resolution. Subjects were seated 52 cm away from the display in an otherwise dark room. Their head was restrained with a chin rest. After completion of the eye position calibration procedure, subjects performed 120 training trials, with RSVP rate set at 400 ms per image rather than 200 ms, until performance reached a level of 80% correct responses. 320 additional testing trials were then performed at 200 ms per image. The instruction given to the subjects was as follows: ‘A small central dot will appear on the screen. You will have to fixate this point throughout the trial. On each trial, a first stream of stimuli will appear at a given location rapidly followed by a second stream of stimuli placed symmetrical to it with respect to the fixation point in the two-hemifields configuration or with respect to the horizontal meridian in the one-hemifield configuration. A cue, embedded in the first stream will tell you in what stream the target is most likely to appear. If the cue is red, then the target will appear in the second stream. If the cue is green, then the target will appear in the first stream. You will have to focus your attention on the first stream in order to correctly identify the cue. The cue correctly predicts the location of the target in 80% of the trials. Your task will be to press on the response button as soon as you detect this target (the target is shown to the subjects). You will first go through a training session, then after a pause, you will be able to go through the main testing session.’

### Data Analysis

Reaction times and detection rates were calculated from subject's responses, where reaction times are taken as the time between target onset and key press and detection rates as the ratio between the number of correct trials and the total number of trials. Trials with reaction times smaller than 200 ms were considered as anticipations and excluded from the analysis, on the basis of Go/NoGo studies which show that in their context, reaction times cannot be produced faster than 250 ms [19], [20]. Mean reaction times and mean detection rates were then calculated for each subject and analyzed as a function of cue instruction, target validity and CTOAs using multi-way ANOVAs with repeated measures.

## Results

Except when mentioned otherwise, three-way repeated-measures ANOVAs (target validity×cue instruction×cue to target asynchrony) were performed on mean reaction times and mean detection rates, separately for the two-hemifields and the one-hemifield configurations.

### Validity effects

We first establish that the cueing procedure embedded in the RSVP stream had the intended attention-orienting effects by comparing detection performance on the validly and invalidly cued targets.

#### Two-hemifields configuration

Reaction times (RT) are on average shorter (F(1,6) = 33.4, p = 0.00129) and detection rates higher (F(1,9) = 51.7, p = 0.00005) during valid trials than during invalid trials. These effects are observed for both shift and no-shift cues (table 1A). Reaction times are on average 137 ms shorter during valid shift instruction trials than during invalid shift instruction trials (Duncan post-hoc test, p = 0.0076) and 127 ms shorter during valid stay instruction trials than during invalid stay instruction trials (Duncan post-hoc test, p = 0.0049). Detection rates are 32% higher during valid shift instruction trials than during invalid shift instruction trials (Duncan post-hoc test, p = 0.000076) and 25% higher during valid stay instruction trials than during invalid stay instruction trials (Duncan post-hoc test, p = 0.000218). There is no validity×cue type interaction (F(1,9) = 0.196, p = 0.67 for reaction times or F(1,9) = 0.97, p = 0.35 for detection rates).

**Table 1:** Invalidity effects as a function of cue identity in the two-hemifields configuration and in the one-hemifield configuration.

*configuration*	Cue type	Validity	mean reaction times+/−s.d.	mean detection rates+/−s.d.
*Two-hemifields*	Shift	Valid	483 ms+/−12	82%+/−3
		Invalid	620+/−27	53%+/−6
	Stay	Valid	471 ms+/−14	80%+/−3
		Invalid	598+/−24	55%+/−6
*One-hemifield*	Shift	Valid	547 ms+/−28	73%+/−3
		Invalid	629+/−45	41%+/−9
	Stay	Valid	534 ms+/−20	76%+/−4
		Invalid	615+/−46	45%+/−8

Mean reaction times and detection rates as well as standard errors are presented. All valid versus invalid first order comparisons are significant (p<0.01) while validity×cue interactions are not.

#### One-hemifield configuration

**Figure 3:**
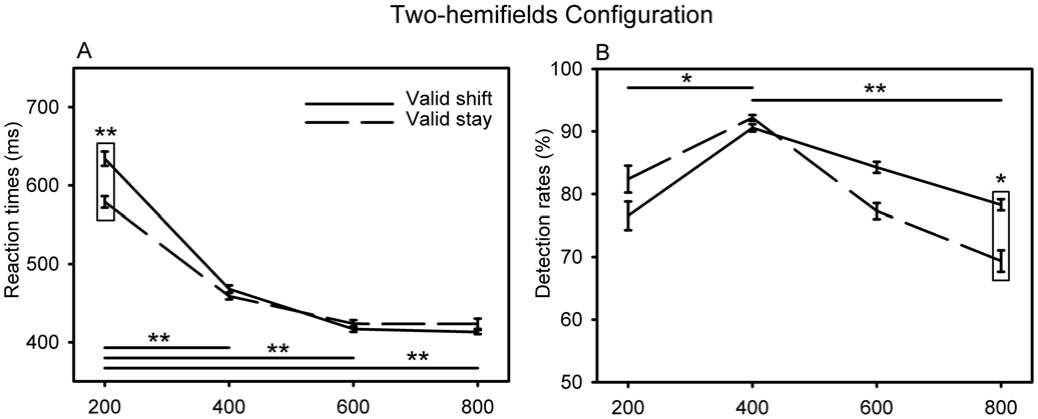
Cue to target onset asynchrony effects in the two-hemifields configuration on (A) mean reaction times and (B) mean detection rates, as a function of cue instruction. Vertical bars represent standard error. **represents p values<0.01 and *represents p values<0.05 on Duncan post-hoc tests. Rectangles represent the comparison between valid shift and valid stay conditions.

In this configuration, the performance advantage of validly cued trials over invalidly cued trials is significant for detection rates (F(1,7) = 11.53, p = 0.008, figure 3B), but just fails to reach significance for reaction times (F(1, 6) = 5.27, p = 0.0614, table 1B). As in the two-hemifields configuration, the validity effect is found for both cue instruction conditions (no validity×cue interaction, F(1,7) = 0.002, p = 0.96). Indeed, detection performance is 32% higher during valid shift instruction trials than during invalid shift instruction trials (Duncan post-hoc test, p = 0.000093) and 31% higher during valid stay instruction trials than during invalid stay instruction trials (Duncan post-hoc test, p = 0.00092).

### Reaction times variability as a function of cue instruction validity

The valid/invalid differences in detection performance (% of trials in which subjects report the presence of a target) is a strong indicator that endogenous orienting of attention enhances, as expected, visual processing of the target. However, it is possible that subjects responded on some trials even if the target went undetected. To address this issue, further information about detection performance can be obtained by examining RT variability. If the target cannot be detected by the subjects, then on those trials in which it is reported nevertheless, RTs are expected to follow a uniform distribution around target onset. Provided that enough trials are available for the analysis, this assumption is not expected to be affected by whether subjects are forced to respond on all trials or not. In order to quantify the degree to which the target is perceived as a function of cue instruction, the following analysis was carried out. For each subject, as many simulated reaction times as the actual number of trials available for the given condition were drawn from uniform distributions bounded by the minimum and maximum RTs in the condition of interest. This was repeated 1000 times and each time, a simulated RTs standard deviation was generated. These values were averaged to yield an average simulated RTs standard deviation per subject and plotted against the actual observed RTs standard deviation (figure 2). In the two-hemifields configuration, observed and simulated RTs are significantly different both in the valid condition (t-test, p<0.0001) and in the invalid condition (p = 0.03). Note that in spite of these statistically significant differences, RTs distribution in the invalid condition is close to the random range, indicating a somewhat weak relationship between manual RTs and target onset. In the one-hemifield condition, observed RTs standard deviation is significantly different from simulated RTs variance in the valid condition (p = 0.01) but not in the invalid condition (p = 0.2). Thus, RTs standard deviation is higher on invalidly than validly cued trials, suggesting that targets were often undetected and that subjects responded at random on invalid trials. This effect is particularly marked in the one-hemifield condition, where observed RTs standard deviation is indistinguishable from random standard deviation. These observations mitigate the relevance of RTs as a measure of performance for invalidly cued trials in the one hemifield configuration, as well as hint of possible differences in attentional control within and across hemifields.

### Cue to target onset asynchrony effects and temporal dynamics

The foregoing analyses concern the effects of cueing on performance as a function of the interval between cue and target. We do not consider invalid cue trials because their small number precludes statistical analyses and also because the high values of individual standard deviation described above cast serious doubts on the meaningfulness of RT as a measure of performance on invalid cue trials.

#### Two-hemifields configuration

Effects of CTOA and cue instruction were assessed by means of a two-way cue×CTOA anova. Target detection performance was found to depend upon the CTOA (RT F(3,27) = 3.98, p = 0.018, detection rate F(3,27) = 4.66, p = 0.009). This is essentially characterized by longer reaction times for the shortest (200 ms) CTOA as compared to longer ones (Figure 3A) and by optimal detection rates on the 400 ms CTOA (Figure 3B). Cue instruction main effects were not significant (RT F(1,9) = 1.65, p = 0.23, detection rate (F(1,9) = 1.87, p = 0.20) but the cue instruction×CTOA interaction effects were significant (RT F(3,27) = 3.98, p = 0.018, detection rate F(3,27) = 4,66, p = 0.0094). A Duncan post-hoc analysis reveals that this interaction effect is essentially due to a larger early RT cost for shift than for the no-shift cue (p = 0.0013 on the 200 ms CTOA, light box on figure 3A). It takes 55 ms longer to shift attention to the other hemifield than to maintain on its current location. For detection rates, the interaction effect essentially reflects a longer lasting target detection advantage for the shift than for the no-shift cue (p = 0.016 on the 800 ms CTOA, [shift detection rate] – [no-shift detection rate]  = 9%, light box on figure 3B).

#### One-hemifield configuration

**Figure 4:**
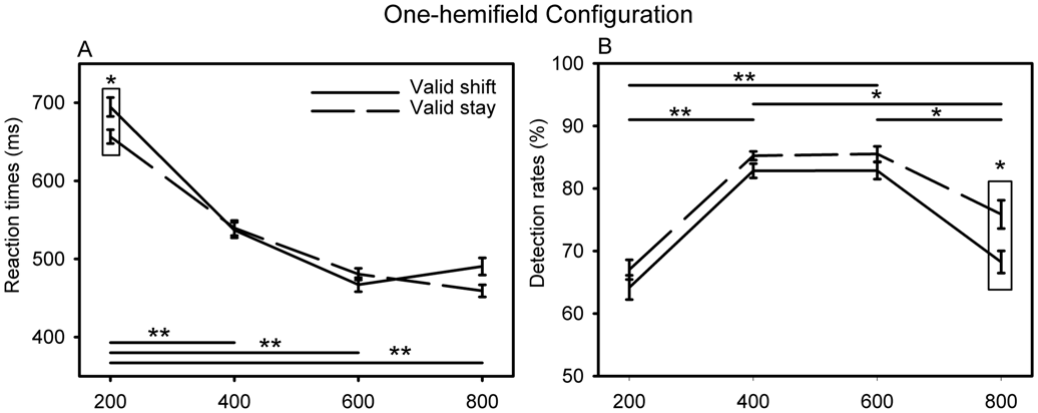
Cue to target onset asynchrony effects in the one-hemifield configuration on (A) mean reaction times and (B) mean detection rates, as a function of cue instruction. All as in figure 3.

Performance also varies as a function of the CTOA in the one-hemifield configuration (RT F(3,27) = 28.8, p<0.0001, figure 4A, detection rate F(3,27) = 5.48, p =  0.0044, figure 4B). This is again essentially due to longer (up to 240 ms) reaction times at the earliest CTOA as compared to later CTOAs and to higher detection rates (up to 20%) on the middle CTOAs as compared to the early and late ones.

Unlike in the two-hemifields configuration, no statistically significant interaction between the CTOA and the cue instruction can be seen both for reaction times (F(3,27) = 2.01, p = 0.135) and for detection rates (F(3,27) = 0.48, p = 0.69). In spite of this, there is a statistically significant early cue instruction effect on reaction times which are 38 ms longer on shift trials than on stay trials (Duncan test, p = 0.041). A significant late cue instruction effect can also be noted on detection rates which are 8% higher on stay trials than on shift trials (Duncan test, p = 0.044).

## Discussion

The aim of the present study was to assess the spatial and temporal dynamics of voluntary attention shifts and re-engagement, both in the same hemifield and across hemifields. In a dual peripheral stream RSVP task, subjects were cued to maintain (re-engage) their attention in the currently attended spatial location or to shift it to another location. These cues were predictive of target location in 80% of the trials. Subjects' detection performance on invalid trials was significantly lower than on valid trials, with reaction time distributions that were nearly or completely indistinguishable from those predicted from a random response hypothesis, indicating that cue instruction was used by the subjects to orient their attention [21].

### How long does it take voluntary attention to shift from one point to another

The temporal dynamics of attention has been investigated by Sperling and his collaborators in the 80's using a dual stream RSVP paradigm [22], [23]. As seen in the introduction, the authors report attention reaction times in the range of 300 to 400 ms. This time estimate includes the time needed to switch one's attention as well as the time needed to shift from analyzing numerals to analyzing letters and to recognize the target letter. This confound still holds true for all the more recent studies on voluntary attention reaction times.

In our study, subjects were required to maintain their attention at a given location or to shift it to another location in the visual field. If we consider only the 200 ms CTOAs, a mean reaction time can be estimated both in the shift condition (634 ms for the across hemifields configuration and 694 ms for the within hemifield configuration) and in the stay condition (579 ms and 656 resp.). Both these reaction times can be decomposed as follows:

***On*** **shift** ***cue reaction time***  =  *shift* cue interpretation + attention shift time + attentional engagement + motor execution

***On*** **stay** ***cue reaction time***  =  *stay* cue interpretation + attentional engagement + motor execution

Note that the attention reaction time of Sperling and collaborators corresponds to cue interpretation+attention shift time+a bottleneck process involving both digit detection and memorization. Here, the shift and stay cues were homogeneous patches differing only in hue and luminance, we thus assume that the time for the subjects to perceive and process each one is the same. However, whereas the shift cue instructs the subjects to move their attention to a given location, the stay cue instructs the subjects to keep attending the same location. Thus, the difference between the shift cue and stay cue reaction times can be considered a good estimate of the pure attention shift time on the shift conditions. This gives an average estimate of 38 ms for within hemifield shifts and 55 ms for across hemifields shifts. One could argue that the difference of luminance of the cues could affect the reaction times [17]. However, this difference seems not to be sufficient to generate significant differences in subject's reaction times according to the chromatic contrast between the two cues [18].

We see two potential reasons for these different within and across hemifields estimates. First, there could be a distance effect since, although the retinal eccentricity radius of the stimuli were the same in the two configurations (i.e. 10°), the Euclidian distance between the two streams was shorter in the one-hemifield (14°) than in the two hemifields (20°) configuration. Hazlett et al. (2004) showed that attentional shift times contain two components: a planning phase that is dependent on the spatial extent of the attentional shift to be prepared and an execution phase that is independent of it. In our task, on CTOA200, planning and execution stages cannot be dissociated. Thus the shift time difference between the within and across hemifields configuration described here could be in part due to the distance effect described by Hazlett et al. Alternatively, this shift time difference could be due to intrinsic processing differences between the two configurations such as interhemispheric transfer delays, or to more complex visual integration processes. The spatial configuration used by Hazlett et al. does not provide elements on this question as their protocol involved only within hemifield attentional shifts. Further experiments will need to be carried out in order to clarify this issue.

The shortest cue to target interval we have tested is of 200 ms. It could be argued that our estimates of attentional shift times are a lower bound of the actual values, and that shorter CTOAs would have given us higher values. Two arguments can be opposed to this. First, attention reengagement is a time consuming process even when no spatial shift is needed, as demonstrated by the fact that detection rates rise from CTOA200 to CTOA400. Second, our estimates are very close to the 50 ms minimal dwell time of attention on visual items during whole field visual search [13], [24], [25]. This estimate of dwell time includes both the perceptual analysis of stimuli and the attentional serial shift, averaged over both across and within search paths. To our knowledge, there are no visual search estimates of attention dwell time that precisely address the question of across and within hemifield attentional dwell time differences.

Thus we argue that attentional shifting is a time-consuming process which can be estimated, with the present approach, independently from other sensory, cognitive or motor variables. Interestingly, the values we report here is close to the 50 ms attention shift time reported for exogenous attentional shifts following for example the flash of visual stimulus [26]–[28].

### The temporal deployment of attention

The above discussion dealt with the timing of attention shifting, that is the moment at which attentional resources become significant enough at the new location to affect either reaction times or detection rates. A related issue is how these resources unfold in time and whether this process depends or not on attentional instructions and on the spatial layout of the stimuli. In several studies, a valid endogenous cue is shown to be virtually invalid at 100 ms from its onset (affecting performance negatively) and to increase its benefit steadily until 400 ms [for example, 7]. In the present study, the maximum of performance is also obtained 400 ms after cue onset. Interestingly, major differences are seen between the within-hemifield and across-hemifields configurations. Indeed, while in the across-hemifields configuration, after an initial increase, performance decays after 400 ms, in the within-hemifields configuration, this decay is delayed after 600 ms. One possible explanation for this difference is that attention takes longer to disengage in the within hemifields configuration and would be revealed by a higher temporal resolution. Alternatively, it could be that the engagement-disengagement process starts earlier in time in the across hemifields configuration than in the within hemifields configuration. Rerunning the experiments with faster image rates would help disambiguate these two possibilities.

Another striking difference is that while in the across hemifields configuration, the temporal dynamics of attentional unfolding is very similar between shift and stay conditions if corrected for the delay due to the attentional shift, in the within hemifields configuration, attention seems to stay engaged longer following a shift instruction as compared to a stay instruction, as revealed by the detection rates on the longest CTOA. This suggests that attentional engagement/re-engagement processes may be different across and within hemifields.

### Divide or not divide?

The analysis of reaction times distributions shows that, in the across hemifields configuration, this measure is significantly different from a uniform distribution both in the valid and invalid trials, meaning that subjects consistently detect the target both on the spatial position where it is expected and on the other spatial position where the target appears in less than a fifth of the trials. This implies that in this configuration, attentional resources can be divided between the two spatial locations. In contrast, in the one-hemifield configuration, reaction times distribution is significantly different from the uniform distribution only in the valid trials, meaning that subjects consistently detect the target on the spatial position where it is expected, but are unable to do so on the other spatial position. This suggests that in this spatial configuration, the attentional resources are mostly allocated on the cued spatial position.

Several factors can be proposed to account for this differential ability to split attention across and within hemifields. One of them is that visual acuity is higher along the horizontal than the vertical axis [29]–[31]. However, Beirne et al. show that there is no difference between horizontal and vertical acuity for eccentricities of 10°, which corresponds to the eccentricity at which our stimuli are presented in both configurations. Another factor is differences in lateral inhibition, as the distance between the two spatial positions of interest is bigger in the across configuration (20°) than in the within configuration (14°), resulting in a higher competition between the two visual streams in the within hemifields configuration [32]–[34]. Although this explanation cannot be disregarded, such low-level sensory-sensory competition components are not expected at those distances. A third factor that may have contributed to these effects is task difficulty. A recent study by Kraft et al. [35] shows that in a peripheral discrimination task, performance is always better when the stimuli are presented across hemifields than within. Here, even though the distance between the two spatial positions of interest is bigger in the across-hemifields configuration (20°) than in the within-hemifield configuration (14°), as pointed out previously, average detection rates on valid trials are higher in the former (81%) than in latter (76%) configuration, confirming the relative performance advantage afforded by having to attend to simultaneous stimuli presented in different hemifields as reported by Kraft et al. Other studies have shown that splitting of the attentional spotlight is possible both in across hemifield configurations [36]–[38] and in within hemifield configurations [38]. The fact that we failed to observe it in our within-hemifield configuration could imply that it is intrinsically more difficult to split attention within than across hemifields and that attending to two locations at the same time is only possible if the task demands at the primary location do not exceed a certain level. Only electrophysiological or high resolution imaging studies can address this question by asking how attentional neuronal substrates encoding each stream are recruited as a function of the across or within hemifield configuration.

### Conclusion

In conclusion, by the use of a new paradigm we have estimated the time cost of voluntary spatial attention shift at 55 ms for across hemifields shifts and at 38 ms for within hemifields shifts. We also provide evidence suggesting that within hemifield spotlight division is more difficult to operate than across hemifields spotlight suggesting that across and within hemifields attentional orientation may operate under different functional constraints.
